# Stability of Anthocyanins and Their Degradation Products from Cabernet Sauvignon Red Wine under Gastrointestinal pH and Temperature Conditions

**DOI:** 10.3390/molecules23020354

**Published:** 2018-02-07

**Authors:** Ping Yang, Chunlong Yuan, Hua Wang, Fuliang Han, Yangjie Liu, Lin Wang, Yang Liu

**Affiliations:** 1 College of Enology, Northwest A&F University, Yangling 712100, China; duolamiwawa@126.com (P.Y.); yuanchunlong@hotmail.com (C.Y.); j1349266163@163.com (Y.L.); maggie_wongkaixin@163.com (L.W.); pjxy122ly@163.com (Y.L.); 2 Shaanxi Engineering Research Center for Viti-Viniculture, Northwest A&F University, Yangling 712100, China; 3 Heyang Viticulture Experimental Station, Northwest A&F University, Heyang 715300, China

**Keywords:** Cabernet Sauvignon wine, anthocyanins, degradation, stability, gastrointestinal pH and temperature

## Abstract

This study investigated the stability of wine anthocyanins under simulated gastrointestinal pH and temperature conditions, and further studied the evolution of anthocyanin degradation products through simulated digestive conditions. The aim of this study was to investigate the relation between anthocyanins’ structure and their digestive stability. Results showed that a total of 22 anthocyanins were identified in wine and most of these anthocyanins remained stable under simulated gastric digestion process. However, a dramatic concentration decrease happened to these anthocyanins during simulated intestinal digestion. The stability of anthocyanins in digestive process appeared to be related to their structure. The methoxy group in the B-ring enhanced the stability of anthocyanins, whereas hydroxyl group resulted in a reduction of their stability. Acylation decreased the stability of malvidin 3-*O*-glucoside. Pyruvic acid conjugation enhanced the structural stability of pyranoanthocyanins, whereas acetaldehyde attachment weakened their stability. A commercial malvidin 3-*O*-glucoside standard was used to investigate anthocyanin degradation products under simulated digestion process, and syringic acid, protocatechuic acid and vanillic acid were confirmed to be the degradation products via anthocyanin chalcone conversion path. Gallic acid, protocatechuic acid, vanillic acid, syringic acid, and *p*-coumaric acid in wine experienced a significant concentration decrease during digestion process. However, wine model solution revealed that phenolic acids remained stable under gastrointestinal conditions, except gallic acid.

## 1. Introduction

Anthocyanins are the major colorants in grapes and wine, and they have been confirmed to play important roles in affecting the appearance and sensory attributes of grapes and wine [1,2,3,4,5]. More importantly, anthocyanins can serve as one of the most important antioxidants in grapes and wine to provide multiple health promoting properties [6,7,8]. For example, it has been reported that anthocyanins possess the anti-cancer, anti-inflammatory, antimicrobial features, and the consumption of these antioxidants can lower the incidence of cardiovascular, diabetic, and obesity diseases [9,10,11,12,13,14,15].

**Figure 1:**
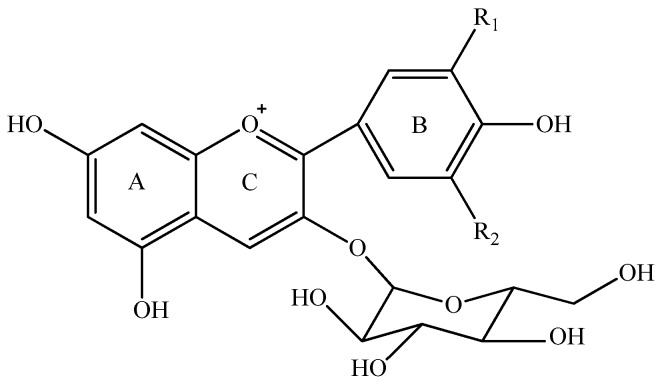
Five monomeric anthocyanins in red wine of *Vitis Vinifera* L.

Anthocyanins can be synthesized as secondary metabolites in grapes during the grape development period [16,17]. These colorants can be extracted into red wine during the wine maceration process and they can further be metabolized during the wine fermentation and aging period [1,18,19]. Therefore, the composition and concentration of anthocyanins mainly determine the appearance of red wine. It has been reported that the level of anthocyanins in red wine ranges from 10 to 2000 mg/L [19,20,21], and their level in wine is mainly determined by grape variety, climate conditions, management system, and fermentation technique [3,19,22,23,24,25,26]. Regarding their chemical nature, anthocyanins can be divided into monomeric anthocyanins (Figure 1), acylated anthocyanins, pyranoanthocyanins, and polymeric anthocyanins [27,28,29,30,31]. In *Vitis vinifera* grapes and wine, the major monomeric anthocyanins include pelargonidin 3-*O*-glucoside, delphinidin 3-*O*-glucoside, cyanidin 3-*O*-glucoside, petunidin 3-*O*-glucoside, peonidin 3-*O*-glucoside, and malvidin 3-*O*-glucoside (Figure 1) [2,26,31]. The monomeric anthocyanins can be further converted into the acetylated, coumaroylated, and caffeoylated anthocyanins [2,30,31]. Pyranoanthocyanins can be synthesized through conjugating monomeric or acylated anthocyanins with low molecular compounds in wine, whereas the interaction among anthocyanins or between anthocyanins and other phenolic compounds could result in the formation of polymeric anthocyanins [30,31,32,33]. Pyranoanthocyanins and polymeric anthocyanins normally are formed during wine aging process, which could shift wine color from bright red color to brick and dark red color [34,35].

It has been reported that anthocyanins, like other phenolic compounds, can be absorbed through the human gastrointestinal tract. The absorption rate of anthocyanins is reported to be around 0.1% to 2%, and they could be readily distributed to different organs, including liver, lung, kidney, prostate, heart, and brain [6,36,37,38]. The major digestive segments in the human gastrointestinal tract include stomach and small intestine. Nutrient digestion mainly happens in the stomach, whereas the small intestine is the major place where nutrients are absorbed [39,40]. It has been reported that 10% to 25% of anthocyanins were degraded in stomach, whereas the small intestine was responsible for 30% to 50% of the anthocyanins’ degradation [41,42,43,44,45]. Anthocyanin degradation can happen at their A- and/or B-ring. The A-ring degradation has been reported to result in the formation of formylphloroglucinal or phloroglucinol carboxylic acid, whereas syringic, vanillic and protocatechuic acids were the major products from the anthocyanins degradation via B-ring cleavage [46,47,48]. 

It has been accepted that the percentage of anthocyanins’ degradation in the human gastrointestinal tract is closely correlated to the anthocyanins’ structure. However, such investigations have not been well documented to our best knowledge, especially regarding the acylated anthocyanins, pyranoanthocyanins, and polymeric anthocyanins found in wine. Therefore, the present study selected anthocyanins in Cabernet Sauvignon wine, and further investigated their stability under simulated human gastrointestinal pH and temperature conditions. Furthermore, anthocyanin standards and their potential degradation products were also investigated under the simulated gastrointestinal conditions to elucidate the degradation pathway of anthocyanins. The findings from this study could provide useful information on the elucidation of the anthocyanins’ stability and metabolism in the human gastrointestinal tract.

## 2. Results and Discussion

### 2.1. Anthocyanins and Phenolic Acids in Red Wine

**Figure 2:**
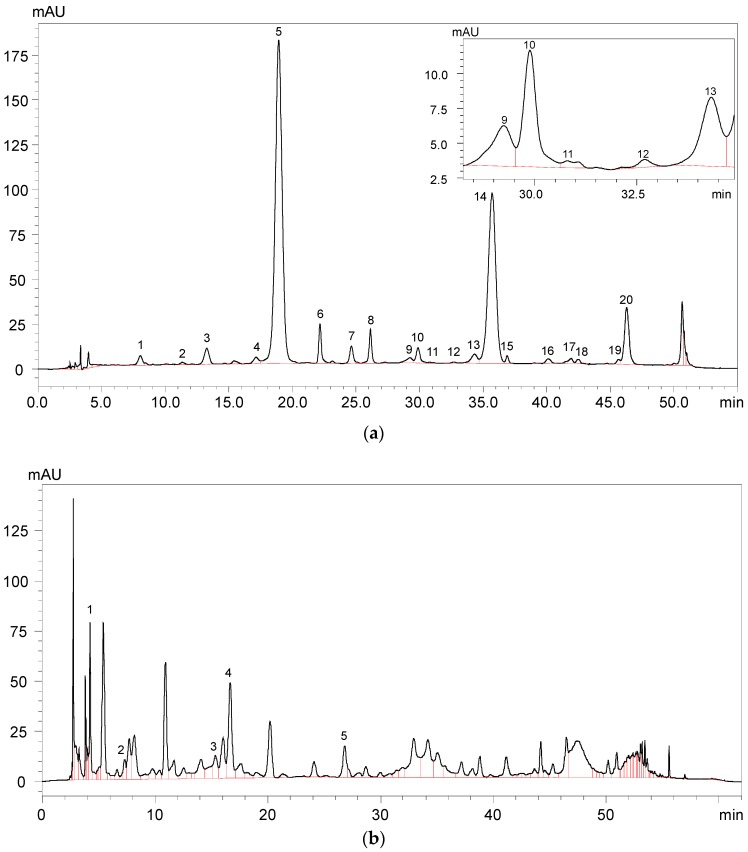
HPLC chromatography of (**a**) identified anthocyanins and (**b**) phenolic acids in Cabernet Sauvignon red wine.

A total of 22 anthocyanins were identified in the wine samples, including five monomeric anthocyanins, six acylated anthocyanins, eight pyranoanthocyanins, and three polymeric anthocyanins (Figure 2a and Table 1).

It should be noted that malvidin 3-*O*-glucoside-4-vinylphenol adduct and malvidin 3-*O*-(6-*O*-acetyl)-glucoside-4-vinylphenol adduct were not quantified due to their limited concentration in the wine sample. The total anthocyanins concentration in the wine sample was about 299.75 mg/L (Table 1). Malvidin 3-*O*-glucoside appeared to be the highest individual anthocyanin in the wine sample and its concentration represented more than 49% of the total anthocyanin concentration. Besides, malvidin 3-*O*-(6-*O*-acetyl)-glucoside and malvidin 3-*O*-(6-*O*-*trans*-*p*-coumaryl)-glucoside were also found to be the predominant anthocyanins in the wine sample, and their concentration accounted for about 29% and 6% of the total anthocyanins content, respectively. Gallic acid, protocatechuic acid, vanillic acid, syringic acid, and *p*-coumaric acid were all detected in the wine sample (Figure 2b and Table 2). It should be noted that syringic acid exhibited the highest concentration in the wine sample, whereas *p*-coumaric acid existed in the wine with the lowest concentration.

### 2.2. Evolution of Anthocyanins and Phenolic Acids under Simulated Gastrointestinal pH and Temperature Conditions

#### 2.2.1. Total Anthocyanins

**Figure 3:**
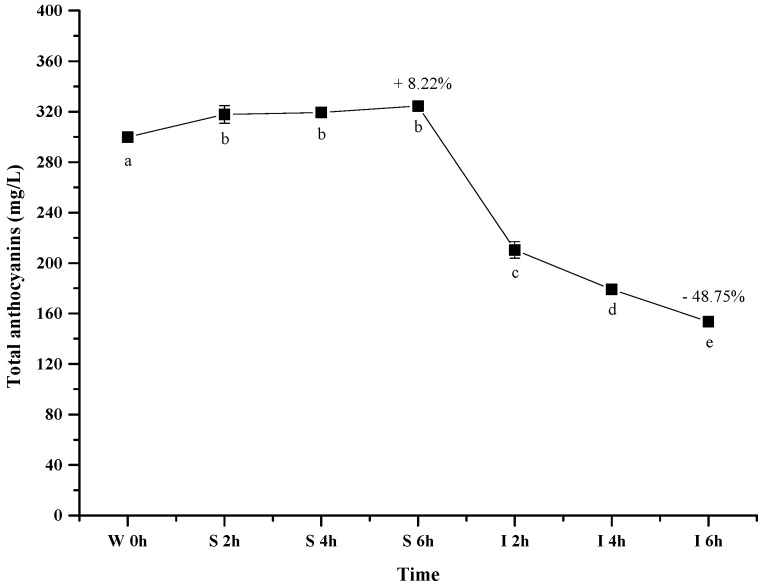
Content alteration of total anthocyanins in red wine during simulated digestion process (wine sample: W 0 h; S 2 h, S 4 h, S 6 h: samples under the gastric condition; I 2 h, I 4 h, I 6 h: samples under the intestinal condition; different lowercase letter means significant difference at 0.05 level).

Figure 3 shows the evolution of the total wine anthocyanins in the simulated gastrointestinal pH and temperature conditions. It was observed that incubating the wine sample in the simulated gastric condition significantly resulted in an apparent 8.22% increase of the total anthocyanin concentration after 6 h (*p* < 0.05). However, its concentration decreased significantly by about 49% after digesting the wine in the simulated intestinal pH condition (after 12 h) (*p* < 0.05). Our result was consistent with the previous report [44]. 

It has been known that anthocyanin molecules possess an equilibrium under different pH conditions, and lower pH environment could result most anthocyanins in the flavylium cation conformation [49]. Such a conformation shift led to an apparent increase on the total anthocyanins concentration under the simulated gastric condition. In the simulated intestinal tract, the decrease of the total anthocyanins concentration mainly resulted from the degradation of anthocyanins via cleaving their aromatic ring as suggested from previously studies [47,50]. Additionally, basic condition in the simulated intestinal tract was also reported to trigger the interactions between anthocyanin molecules and other compounds to yield polymeric compounds [43,51]. Such interactions could also result in the decrease of the total anthocyanins concentration.

#### 2.2.2. Monomeric Anthocyanins

**Figure 4:**
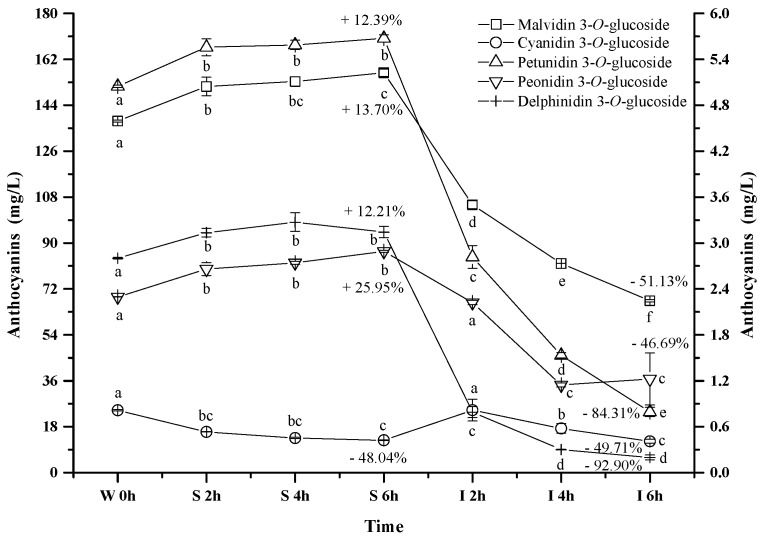
Content alteration of monomeric anthocyanins in red wine during simulated digestion process (Left axis: malvidin 3-*O*-glucoside; right axis: other anthocyanins; wine sample: W 0 h; S 2 h, S 4 h, S 6 h: samples under the gastric condition; I 2 h, I 4 h, I 6 h: samples under the intestinal condition; different lowercase letter means significant difference at 0.05 level).

Five monomeric anthocyanins (Figure 1), including malvidin 3-*O*-glucoside, petunidin 3-*O*-glucoside, peonidin 3-*O*-glucoside, delphinidin 3-*O*-glucoside, and cyanidin 3-*O*-glucoside, were detected in the wine sample. Malvidin 3-*O*-glucoside, petunidin 3-*O*-glucoside, peonidin 3-*O*-glucoside, and delphinidin 3-*O*-glucoside exhibited the similar evolution pattern under the simulated gastrointestinal conditions (Figure 4). These four monomeric anthocyanins increased their apparent concentration in the simulated gastric condition, and then significantly decreased their level under the simulated intestinal tract (*p* < 0.05). Our results were in accordance with the previous reports [44,45,52]. Flavylium cation conformation has been confirmed to be the dominant structure for monomeric anthocyanins under the acidic condition, and this conformation can benefit the stability of anthocyanins [49]. This could explain the apparent increase on these monomeric anthocyanins during the incubation at the simulated gastric tract. The basic condition in the simulated intestinal condition could facilitate the cleavage of the C-ring in anthocyanins structure, which led to a concentration decrease of these monomeric anthocyanins [42]. It should be worth noting that cyanidin 3-*O*-glucoside exhibited an opposite evolution during the incubation at the simulated gastrointestinal condition compared to the other monomeric anthocyanins. A significant concentration decrease of cyanidin 3-*O*-glucoside was found under the simulated gastric conditions, whereas incubating the wine under the simulated intestinal condition resulted in a concentration increase and then a decrease of this anthocyanin (*p* < 0.05) (Figure 4). Acidic conditions might cause the release of the sugar moiety from its aglycone due to hydrolysis, whereas flavylium cation conformation could stabilize anthocyanins under acidic conditions [40]. Therefore, we speculated that hydrolysis and/or polymeric reaction might take place more rapidly on cyanidin 3-*O*-glucoside than the formation of its flavylium cation structure, which caused its concentration decrease under the simulated gastric tract [43,51]. During the incubation at the simulated intestinal condition, cyanidin 3-*O*-glucoside exhibited a concentration increase initially, followed by a decrease. Its concentration increase might result from the degradation of polymeric anthocyanins or bound anthocyanins or other unknown reason [51,53]. However, the basic condition eventually caused cyanidin 3-*O*-glucoside to be degraded, which lowered its concentration in the simulated intestinal condition.

It should be noted that after the digestion process the concentrations of delphinidin 3-*O*-glucoside, petunidin 3-*O*-glucoside, malvidin 3-*O*-glucoside, peonidin 3-*O*-glucoside, and cyanidin 3-*O*-glucoside decreased significantly by 92.9%, 84.3%, 51.1%, 49.7% and 46.7%, respectively (*p* < 0.05) (Figure 4). Their stability in the digestive system appeared to be related to their molecular structure. The increase on the substituent groups in the B-ring of anthocyanins resulted in a significant decrease on the stability of the monomeric anthocyanins in the simulated gastrointestinal tract, whereas the methoxy group presence on the B-ring could stabilize the anthocyanins structure in the digestive system [54,55]. It should be aware that more hydroxyl groups in the anthocyanins structure accelerated the degradation of anthocyanins in the digestive system [54,55].

#### 2.2.3. Acylated Anthocyanins

**Figure 5:**
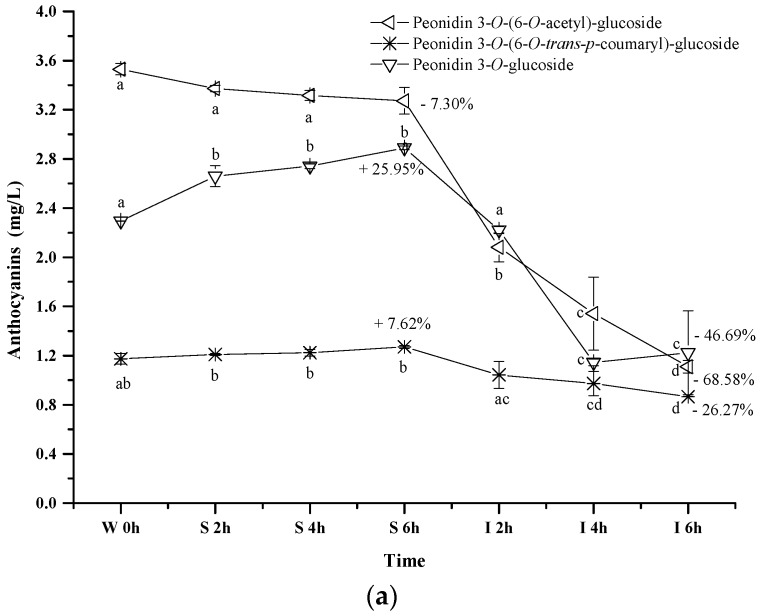

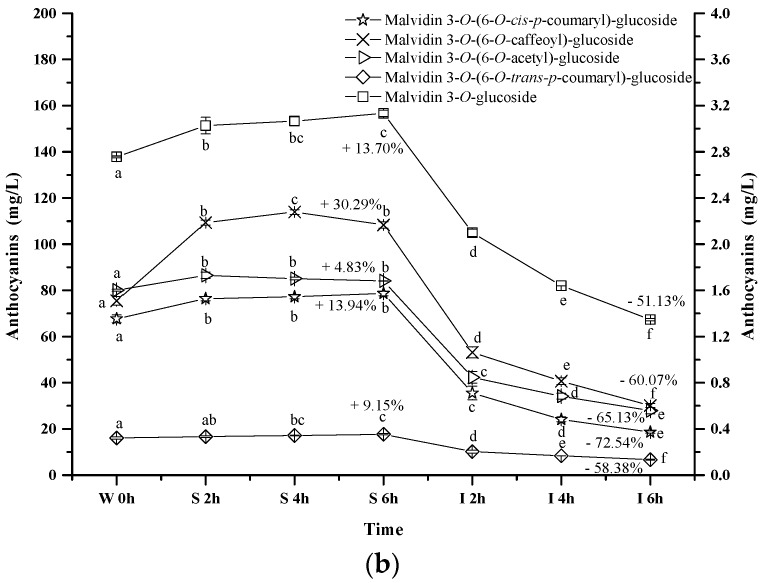
Content alteration of acylated anthocyanins in red wine during simulated digestion process (Left axis: malvidin 3-*O*-(6-*O*-acetyl)-glucoside, malvidin 3-*O*-(6-*O*-*trans-p*-coumaryl)-glucoside, malvidin 3-*O*-glucoside; Right axis: malvidin 3-*O*-(6-*O*-*cis-p*-coumaryl)-glucoside, malvidin 3-*O*-(6-*O*-caffeoyl)-glucoside; wine sample: W 0 h; S 2 h, S 4 h, S 6 h: samples under the gastric condition; I 2 h, I 4 h, I 6 h: samples under the intestinal condition; different lowercase letter means significant difference at 0.05 level).

Peonidin 3-*O*-(6-*O*-*trans-p*-coumaryl)-glucoside exhibited an apparent concentration increase (about 7.62%) under the simulated gastric conditions, whereas the simulated intestinal conditions reduced its concentration by 26.27% (*p* < 0.05) (Figure 5a). Its evolution pattern was similar to that of peonidin 3-*O*-glucoside under the digestive conditions. However, a continuous concentration decrease happened to peonidin 3-*O*-(6-*O*-acetyl)-glucoside during the whole digestion process (*p* < 0.05). For instance, about a 7.3% concentration decrease was observed under the simulated gastric conditions, whereas its concentration decreased by 68.58% after the incubation under the simulated intestinal conditions. These indicated that the presence of an acetyl group in the peonidin 3-*O*-glucoside structure might lower the structural stability under acidic and basic conditions. In addition, peonidin 3-*O*-(6-*O*-acetyl)-glucoside or its chalcone structure might react with other compounds (such as acetaldehyde, pyruvic acid, etc.) to form the new unknown compounds [43,51]. Compared to the acetyl group, the coumaryl group addition stabilized the structure of peonidin 3-*O*-glucoside, leading to a lesser degradation rate under the simulated intestinal conditions.

Similarly, the concentrations of malvidin 3-*O*-(6-*O*-acetyl)-glucoside, malvidin 3-*O*-(6-*O*-*trans-p*-coumaryl)-glucoside, malvidin 3-*O*-(6-*O*-*cis-p*-coumaryl)-glucoside, and malvidin 3-*O*-(6-*O*-caffeoyl)-glucoside apparently increased by 4.83%, 9.15%, 13.94%, and 30.29% after 6 h incubation under the simulated gastric conditions, respectively (*p* < 0.05) (Figure 5b). However, their concentration dramatically decreased by 65.13%, 58.38%, 72.45% and 60.07% under the simulated intestinal conditions, respectively (*p* < 0.05). These results indicated that different substituent groups affected the stability of anthocyanins in the simulated digestive system, and the *trans-p*-coumaryl addition exhibited the best enhancement on the stability of anthocyanin structure, followed by the caffeoyl group, acetyl group, and the least, the *cis*-*p*-coumaryl group.

#### 2.2.4. Pyranoanthocyanins

**Figure 6:**
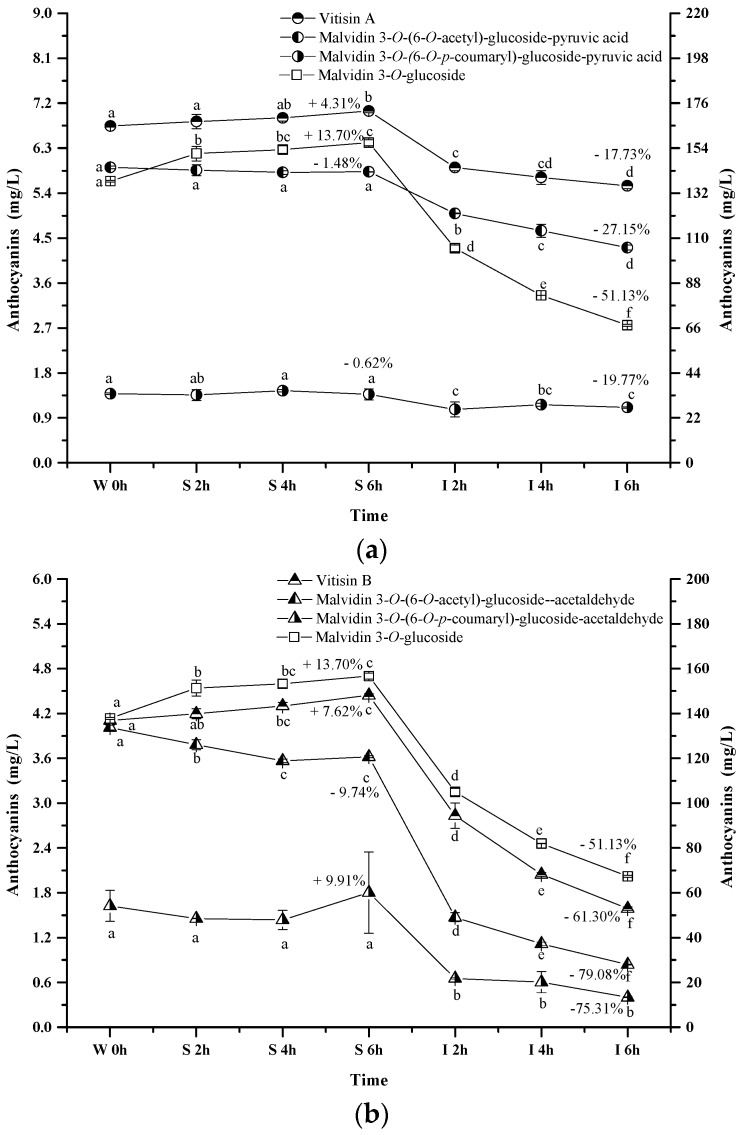
Content alteration of pyranoanthocyanins in red wine during simulated digestion process (wine sample: W 0 h; S 2 h, S 4 h, S 6 h: samples under the gastric condition; I 2 h, I 4 h, I 6 h: samples under the intestinal condition; different lowercase letter means significant difference at 0.05 level).

The pyranoanthocyanins formed in the wine sample of the present study mainly resulted from the interactions through malvidin 3-*O*-glucoside or its acylated anthocyanins with pyruvic acid (Figure 6a). For example, vitisin A is synthesized by conjugating malvidin 3-*O*-glucoside with pyruvic acid. This pyranoanthocyanin significantly increased its apparent concentration in the simulated gastric condition (*p* < 0.05). Acetylvitisin A and coumarylvitisin A after the incubation in the simulated gastric condition remained at similar concentrations, however, their concentration decreased by 17.73%, 19.77% and 27.15% after the incubation under the simulated intestinal conditions, respectively (*p* < 0.05). Compared to the evolution of malvidin 3-*O*-glucoside during the simulated digestion process, the presence of pyruvic acid in the malvidin 3-*O*-glucoside structure enhanced its stability. However, this structural addition reduced the stability of malvidin 3-*O*-(6-*O*-acetyl)-glucoside and malvidin 3-*O*-(6-*O*-*p*-coumaryl)-glucoside under the simulated digestive conditions. Additionally, vitisin B and malvidin 3-*O*-(6-*O*-*p*-coumaryl)-glucoside-acetaldehyde exhibited an apparent concentration increase under the simulated gastric conditions, whereas a concentration decrease was observed in malvidin 3-*O*-(6-*O*-acetyl)-glucoside-acetaldehyde (*p* < 0.05) (Figure 6b). These pyranoanthocyanins experienced a dramatic concentration decrease (61.30%, 79.08%, and 75.31%, respectively) under the simulated intestinal conditions (*p* < 0.05). These indicated that acetaldehyde conjugation weakened the stability of vitisin B and it derivatives. It should be noted that vitisin A and its derivatives were found to be more stable under the simulated digestive condition compared to vitisin B and its derivatives.

#### 2.2.5. Polymeric Anthocyanins

**Figure 7:**
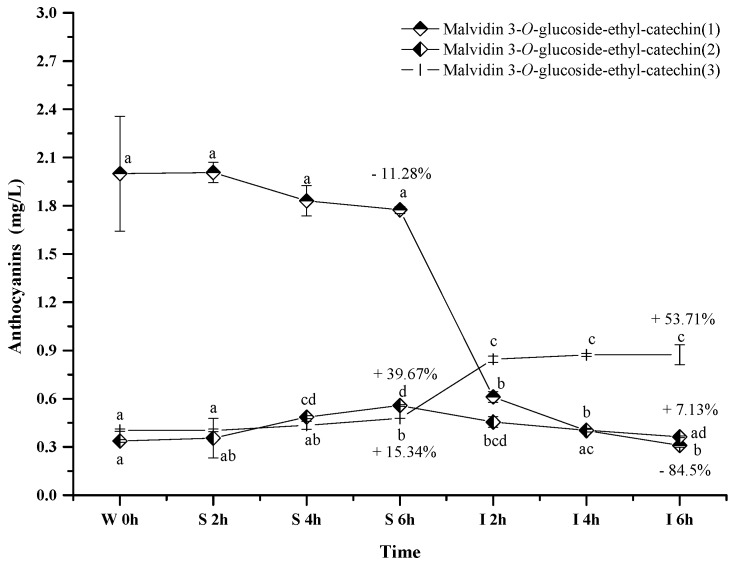
Content alteration of polymeric anthocyanins in red wine during simulated digestion process (wine sample: W 0 h; S 2 h, S 4 h, S 6 h: samples under the gastric condition; I 2 h, I 4 h, I 6 h: samples under the intestinal condition; different lowercase letter means significant difference at 0.05 level).

Only three polymeric anthocyanins, including malvidin 3-*O*-glucoside-ethyl-catechin (**1**), malvidin 3-*O*-glucoside-ethyl-catechin (**2**), and malvidin 3-*O*-glucoside-ethyl-catechin (**3**), were tentatively identified in the wine sample. These polymeric anthocyanins exhibited the different evolution patterns in the simulated digestive condition (Figure 7). Malvidin 3-*O*-glucoside-ethyl-catechin (**1**) showed a decreasing trend on its concentration, whereas an increase happened to malvidin 3-*O*-glucoside-ethyl-catechin (**3**) (*p* < 0.05). Malvidin 3-*O*-glucoside-ethyl-catechin (**2**) showed a concentration increase under the simulated gastric condition but a concentration decrease in the simulated intestinal environment (*p* < 0.05). Under acidic conditions, the concentration increase might result from the reaction between malvidin 3-*O*-glucoside and catechin, whereas the concentration decrease might result from the further reaction between malvidin 3-*O*-glucoside-ethyl-catechin and other compounds in red wine [56,57]. Under alkaline conditions, the concentration variation might be due to their degradations or the degradations of other bigger molecular polymeric anthocyanins [56,57]. The mechanism behind these polymeric anthocyanins evolution needs to be further investigated.

#### 2.2.6. Phenolic Acids

**Figure 8:**
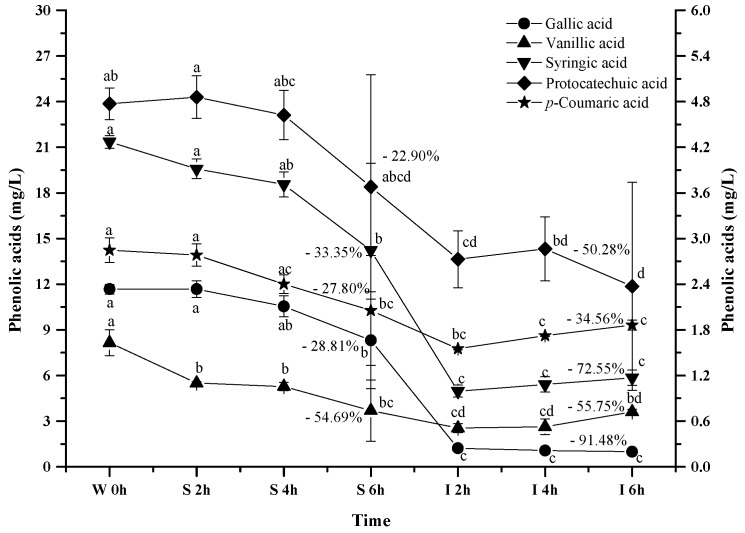
Content alteration of phenolic acids in red wine during simulated digestion process (wine sample: W 0 h; S 2 h, S 4 h, S 6 h: samples under the gastric condition; I 2 h, I 4 h, I 6 h: samples under the intestinal condition; different lowercase letter means significant difference at 0.05 level).

It has been reported that anthocyanins can be degraded into phenolic acids during the digestion process, and gallic acid, protocatechuic acid, vanillic acid, syringic acid and *p*-coumaric acid have been reported to be the major anthocyanins degradation products [46,47,58]. Therefore, we investigated the evolution of these phenolic acids from the wine under the simulated digestion process (Figure 8). 

As the simulated digestion proceeded, an increasing trend on these phenolic acids was expected since most anthocyanins exhibited a dramatic concentration decrease. However, a continuous concentration decrease took place for all the phenolic acids during the digestion process (*p* < 0.05). For example, these phenolic acids decreased their concentration by 28.81%, 22.92%, 54.69%, 33.35%, and 27.80% after the simulated gastric digestion process, respectively (*p* < 0.05). Furthermore, the incubation under the simulated intestinal condition continued to decrease their concentration by 91.4%, 50.28%, 55.75%, 72.55%, and 34.56% (*p* < 0.05). These indicated that these phenolic acids were more stable under acidic conditions than the basic conditions. More importantly, these indicated that the degradation/transformation rate of these phenolic acids was faster than their rate of formation from the anthocyanins’ degradation under the simulated digestion process.

### 2.3. Evolution of Anthocyanin and Phenolic Acid Standards in Simulated Gastrointestinal pH and Temperature Conditions

Wine is a complex system that consists of multiple nutrients and components. To elucidate the relations and degradation mechanism between anthocyanins and phenolic acids in wine, we further conducted the same digestion study using anthocyanin and phenolic acid standards (Table 3).

**Figure A1:**
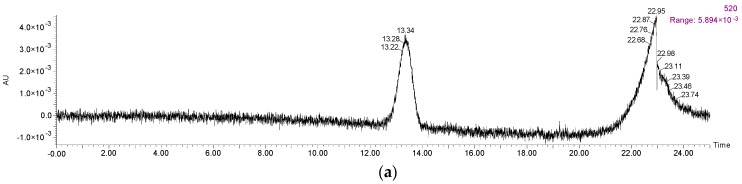

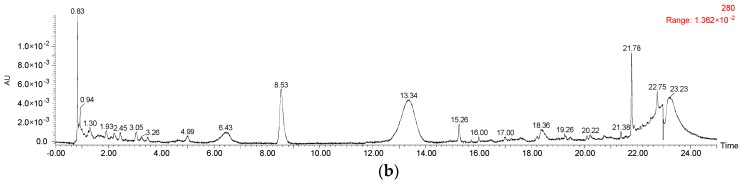
HPLC chromatography of (**a**) malvidin 3-*O*-glucoside standard at 520 nm and (**b**) its degradation products at 280 nm during the simulated digestion process.

**Figure A2:**
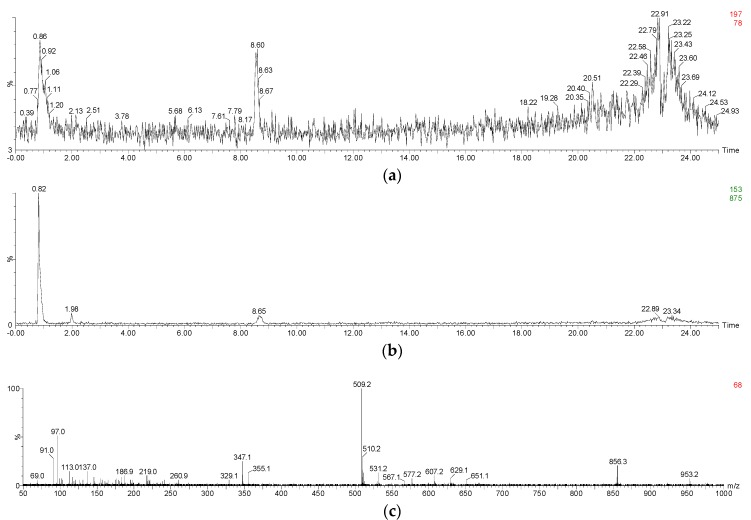
Mass spectrum of (**a**,**b**) syringic acid and ((**c**), negative electrospray ionization mode) malvidin 3-*O*-glucoside-chalcone during the simulated digestion process.

To ensure that phenolic acids are the products of the anthocyanins degradation during the digestion process, a commercial standard of malvidin 3-*O*-glucoside was incubated under the same digestive conditions, and its degradation products were identified using HPLC and UPLC-MS (Table 3 and Appendix A, Figure A1a,b). A tiny amount of peonidin 3-*O*-glucoside was present in the commercial malvidin 3-*O*-glucoside standard ethanol solution. After incubating the standard solution under the simulated digestion conditions, a compound with a precursor ion of *m*/*z* 197 and a product ion of *m*/*z* 153 was detected at extraction ion mode (Appendix A Figure A2a,b). This degradation product was identified as syringic acid. Besides, malvidin 3-*O*-glucoside chalcone was also found due to the presence of its precursor ion of *m*/*z* 509 and product ion of *m*/*z* 347 in negative electrospray ionization mode, and the presence of its precursor ion of *m*/*z* 511 and product ion of *m*/*z* 349 in positive electrospray ionization mode [59,60,61] (Appendix A Figure A2c and Table 3). Similarly, vanillic acid and protocatechuic acid were also identified as the malvidin 3-*O*-glucoside degradation products after the simulated digestion due to their retention time and maximal wavelength feature. 

**Figure 9:**
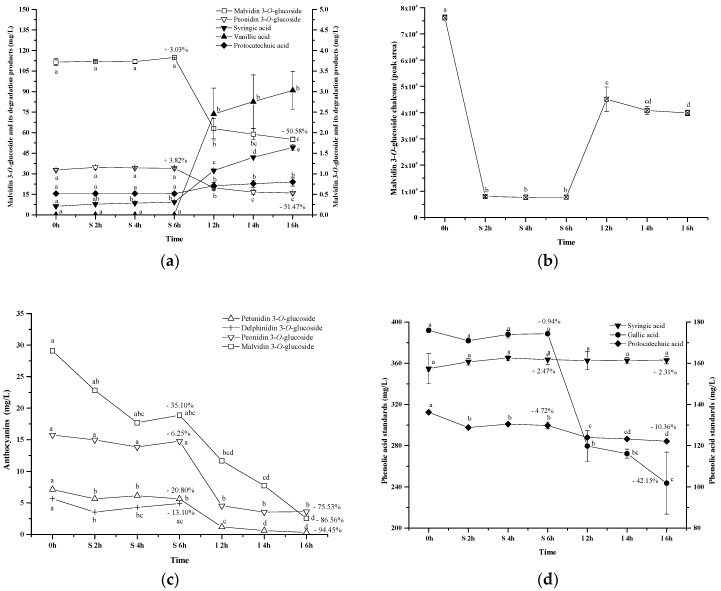
Content alteration of anthocyanin standard (**a**,**c**), its degradation products (**a**,**b**) and phenolic acid standards (**d**) during simulated digestion process ((a): malvidin 3-*O*-glucoside located at left axis, other compounds located at right axis; (b): the content of chalcone malvidin 3-*O*-glucoside was expressed as the peak area; (d): syringic acid located at left axis, protocatechuic acid and gallic acid located at right axis; wine sample: W 0 h; S 2 h, S 4 h, S 6 h: samples under the gastric condition; I 2 h, I 4 h, I 6 h: samples under the intestinal condition; different lowercase letter means significant difference at 0.05 level).

Along with the digestion process, the commercial standards showed the similar concentration during the simulated gastric digestion process. Syringic acid and protocatechuic acid were found with a low level in the solution during the simulated gastric digestion. The simulated intestinal digestion significantly decreased the concentration of the anthocyanins, which resulted in a dramatic concentration increase of syringic acid, protocatechuic acid, and vanillic acid (Figure 9a). Malvidin 3-*O*-glucoside chalcone exhibited a concentration decrease during the simulated gastric digestion process. During the simulated intestinal digestion, its concentration increased and then eventually decreased (Figure 9b). This indicated that chalcone conformation was an intermediate stage during the anthocyanins’ degradation under the digestion process conditions [59]. It should be noted that the anthocyanin standard degradation percentage was not recovered by the formation of these phenolic acids, which was consistent with a previous report [51]. This indicated that further reactions might take place after the formation of phenolic acids. A further study needs to be conducted. Based on the structure of these three phenolics and the five basic anthocyanins (Figure 1), it can be deduced that protocatechuic acid, vanillic acid and syringic acid result from the B-ring of cyanidin 3-*O*-glucoside, peonidin 3-*O*-glucoside and malvidin 3-*O*-glucoside, respectively [46,47,58]. In this study, the commercial standards didn’t contain cyanidin 3-*O*-glucoside. Thus, protocatechuic acid, the degradation product identified tentatively, might result from the other degradation pathway. However, this still needs to be further confirmed.

Additionally, we purified four anthocyanin standards from Yan 73 grape skin, including delphinidin 3-*O*-glucoside, petunidin 3-*O*-glucoside, malvidin 3-*O*-glucoside and peonidin 3-*O*-glucoside. Their purity was 94.7%, 98.1%, 94.3%, and 99.0%, respectively [62]. These anthocyanin standards in the wine model solution exhibited a concentration decrease after the incubation under the simulated gastric conditions (13.10%, 20.81%, 35.10% and 6.25%, respectively). The simulated intestinal conditions significantly decreased their concentration by 100%, 94.45%, 86.56%, and 75.53%, respectively (Figure 9c). These results indicated that peonidin 3-*O*-glucoside was the most stable monomeric anthocyanin, followed by malvidin 3-*O*-glucoside, petunidin 3-*O*-glucoside, and then delphinidin 3-*O*-glucoside. The evolution patterns of these standards were consistent with the evolution of these monomeric anthocyanins from the wine during the digestion process (Figure 4). It should be worth noting that a much higher degradation rate was observed in these monomeric anthocyanin standards compared to those in the wine, indicating that the wine matrix and/or other anthocyanin fractions in the wine might help protect monomeric anthocyanins from degradation under the digestion process conditions.

Syringic acid, protocatechuic acid, or gallic acid standard in the solution was incubated under the same simulated digestive condition to investigate their stability (Figure 9d). Syringic acid appeared to be stable to the digestive conditions. Similarly, the digestion process did not affect the stability of protocatechuic acid and its concentration only decreased by about 10% (*p* < 0.05). Gallic acid remained stable under the simulated gastric conditions. However, a dramatic concentration decrease (42.15%) happened to gallic acid in the simulated intestinal conditions. This result was identical with the previous report [63]. Under basic conditions, gallic acid might be changed into unstable quinone intermediates and/or other resonance forms which may ultimately oxidize in the presence of air to diketo derivatives or other degradation products [63]. However, the degradation mechanism is unclear at present. 

It should be noted that these phenolic acids experienced the significant concentration decrease in the wine during the same simulated digestion process (Figure 8). This indicated that these phenolics (syringic acid, protocatechuic acid, or gallic acid) in the wine might further interact with other compounds (such as anthocyanin, quinone, chalcone, or alcohols), resulting in their concentration decrease [43,51]. 

## 3. Materials and Methods

### 3.1. Evolution of Anthocyanin and Phenolic Acid Standards in Simulated Gastrointestinal pH Conditions

Four anthocyanin standards used for the gastrointestinal condition stability study, including delphinidin 3-*O*-glucoside, petunidin 3-*O*-glucoside, malvidin 3-*O*-glucoside, and peonidin 3-*O*-glucoside, were purified following a published method with some modifications [62]. Briefly, Yan 73 grape skin (5 g) was mixed with 1% HCl *v*/*v* methanol (20 mL). The mixture was sonicated for 30 min at 40 °C, and then centrifuged at 8000 rpm/min for 5 min to collect the supernatant. The residue was then extracted using the same method two more times, and the supernatants were pooled and then filtered through a 0.45 µm membrane filter. The resultant sample was purified using XAD-7HP resin (Amberlite, Sigma-Aldrich, Shanghai, China) and then each anthocyanin fraction was collected using a Shimadzu HPLC system (Shimadzu, Suzhou, China; pump: LC-6AD; diode array detector: SPD-M10-A*VP*; system controller: SCL-10A*VP*) through a fraction collector (FRC-10A). These four anthocyanins had a purity above 94%, and were stored at −20 °C before further study. A commercially available malvidin 3-*O*-glucoside standard (purity: 99.03% with peonidin-3-*O*-glucoside as impurity) was used to investigate the degradation products in the simulated gastrointestinal condition. Phenolic acid standards used for the digestion study included gallic acid, protocatechuic acid, vanillic acid, syringic acid, and *p*-coumaric acid, and these standards were purchased from Sigma-Aldrich.

### 3.2. Chemicals

Formic acid (HPLC grade) was purchased from Kemiou Chemical Reagent Co. Ltd. (Tianjin, China), whereas hydrochloric acid (analytical grade) was purchased from Xilong Chemical Industry Co. Ltd. (Chengdu, China). Ethanol, acetic acid, and sodium hydroxide were obtained from Kelong Chemical Reagents (Chengdu, China). Acetonitrile and methyl alcohol were of HPLC grade and purchased from Tedia Company Inc. (Shanghai, China).

### 3.3. Grape Harvest and Wine-Making

Ripen Cabernet Sauvignon (*Vitis Vinifera* L.) (total soluble solids: 21.5°Brix; titrable acid: 7.6 g/L) grapes were harvested from the Caoxinzhuang vineyard (Yangling, China) in 2015 vintage. The harvested grapes were carefully handpicked and immediately transported back to our laboratory. The grape berries were manually de-stemmed and crushed into juice, and then thoroughly mixed with 50 mg/L SO_2_ in a 10 L glass bottle. After 2 h, 20 mg/L commercial pectinase (Ex-color, Lallemand Inc., Aurillac, France) was added for 24 h. Afterwards, 20 mg/L reactivated yeasts (RV002, Angel, Yichang, China) was inoculated into the juice to initiate wine alcoholic fermentation. During the fermentation process, temperature and must density were monitored three times daily. The fermentation was carried out on the skins at 25 to 28 °C for 7 days. After the pomace was separated, the fermentation continued 2 days. After the alcoholic fermentation, 50 mg/L SO_2_ was further mixed with the wine. The wine had a 12.0% alcohol level, 3.50 g/L reducing sugar content, 6.20 g/L acidity, and 0.48 g/L volatile acidity.

### 3.4. Digestion of Wine Sample, Anthocyanin Standards, and Phenolic Acid Standards in Simulated Gastrointestinal pH and Temperature Contidions

Digestive enzymes were not included in the present simulated gastrointestinal condition since these enzymes have not been reported to digest phenolic compounds [64]. The wine sample was covered with tin foil to ensure dark conditions and was adjusted to pH 1.5 using 0.1% HCl solution. Then, the wine sample was incubated in a 37 °C incubator for up to 6 h. Afterwards, the resultant wine sample was immediately adjusted to pH 7.5 using 1 M NaOH solution and incubated at 37 °C for another 6 h. At each time interval (2 h) during the period of 12 h under the simulated gastrointestinal conditions, each series of wine samples were prepared in duplicate for the analysis of anthocyanins and phenolic acids, and their corresponding marks were labelled W0 as origin wine sample, S 2 h, S 4 h, S 6 h as treatments after 2, 4 and 6 h in simulated stomach conditions, I 2 h, I 4 h and I 6 h as treatments after 2, 4, 6 h in simulated intestinal conditions.

Regarding anthocyanins or phenolic acid standards, each standard was initially dissolved in 12% ethanol solution at pH 3.5. The concentration of malvidin 3-*O*-glucoside standard was 111.48 mg/L with a 1.09 mg/L peonidin-3-*O*-glucoside concentration (impurity). The concentration of syringic acid, protocatechuic acid, and gallic acid were 354.78 mg/L, 136.16 mg/L, and 175.97 mg/L, respectively. The digestion of the standard solution followed the same procedure as the wine sample. Each series of these samples were also prepared in duplicate.

### 3.5. Anthocyanins and Phenolic Acids HPLC Analyses

Anthocyanins and their degradation products were directly analyzed using a Shimadzu LC-20AT HPLC instrument coupled with a photodiode array detector (Shimadzu, Suzhou, China). A Synergi Hydro-RP C18 column (250 × 4.6 mm, 4 µm, Phenomenex, Torrance, CA, USA) was used to separate the anthocyanins in the samples under a flow rate of 1 mL/min. The mobile phase consisted of (A) 2.5% formic acid (*v*/*v*) in water: acetonitrile (8:1, *v*/*v*) and (B) 2.5% formic acid (*v*/*v*) in water: acetonitrile (4:5, *v*/*v*). The gradient was programed as follows: 0 to 45 min, 0% to 35% B; 45 to 46 min, 35% to 100% B; 46 to 50 min, 100% B isocratic; 50 to 51 min, 100% to 0% B; and 51 to 55 min, 0% B isocratic. The wavelength on the photodiode array detector was set at 520 nm. Malvidin 3-*O*-glucoside was used as the external anthocyanin standard for the quantitation of all the anthocyanins in the wine.

Phenolic acids, including gallic acid, protocatechuic acid, vanillic acid, syringic acid, and *p*-coumaric acid were extracted according to a published method [65]. In brief, sample (1 mL) was mixed with ethyl acetate (1 mL) and acetonitrile (0.5 mL) in a 4-mL centrifuge tube. The mixture was vortexed for 10 s and then centrifuged at 5400× *g* for 5 min to collect the organic phase. The sample was extracted two more times with the same extraction procedure. Afterwards, the organic phase extracts were combined and then evaporated under nitrogen stream at room temperature. The dryness was finally dissolved into 0.5 mL methanol for HPLC analysis. A Shimadzu HPLC, equipped with a Synergi Hydro-RP C18 column (250 × 4.6 mm, 4 µm, Phenomenex), was used to analyze the phenolic acids under a flow rate of 1 mL/min. The mobile phase was comprised of (A) 0.1% acetic acid (*v*/*v*) in water: acetonitrile (8:1, *v*/*v*) and (B) 0.1% acetic acid (*v*/*v*) in water: acetonitrile (4:5, *v*/*v*). The gradient was as follows: 0 to 45 min, 0% to 35% B; 45 to 50 min, 35% to 100% B; 50 to 55 min, 100% B isocratic; 55 to 56 min, 100% to 0% B; and 56 to 62 min, 0% B isocratic. Gallic acid, protocatechuic acid, vanillic acid, and syringic acid were detected at 280 nm on the photodiode array detector, whereas *p*-coumaric acid was measured at 320 nm. These phenolic acids were quantified using their corresponding reference standard.

### 3.6. UPLC-ESI-MS/MS

Anthocyanins and their degradation products after digestion in the simulated gastrointestinal tract were identified using a Waters Acquity UPLC system coupled with a BEH C18 column (100 × 2.1 mm, 1.7 µm, Waters Corporation, Milford, MA, USA), a Waters 2489 UV-Visible detector, and a Synapt Q-TOF mass spectrometer (Waters Corporation). The column was maintained at 30 °C and the mobile phase consisted of (A) 5% formic acid (*v*/*v*) in water and (B) 5% formic acid (*v*/*v*) in methanol under a 0.3 mL/min flow rate. The elution gradient was programed as follows: 0 to 30 min, 50% B; 30 to 35 min, 100% B; 35 to 37 min, 0% B. A positive electrospray ion mode was used for the anthocyanins analysis, whereas phenolic acids was ionized under a negative mode with a 3.5 kV capillary voltage, a 20 V cone voltage, and 100 °C dry temperature. A full scan mode from *m*/*z* 50 to 1500 was recorded. Masslynx 4.1 software (Waters Corporation) was used for data collection and analyses.

### 3.7. Statistical Analysis

Data were expressed as the mean ± standard deviation of duplicate tests. The analysis of variance (ANOVA) was carried out using SPSS22.0 software (SPSS Inc., Chicago, IL, USA) under Tukey’s honest significant difference at a significant level of 0.05 (*p* < 0.05).

## 4. Conclusions

In conclusion, most of anthocyanins in wine remained stable under simulated gastric conditions. However, they experienced a dramatic concentration decrease in a simulated intestinal digestion process. Their structure and matrix played an important role in their stability under digestive system conditions. Syringic acid, protocatechuic acid, and vanillic acid were found to result from anthocyanins’ degradation and the anthocyanin chalcone was suggested to serve as an important degradation intermediate to form degradation products. Phenolic acid standards solution remained stable under simulated digestion process conditions. However, a dramatic decrease in their concentration happened to wine during the simulated digestion process.

## Figures and Tables

**Table:** 

Anthocyanins	R_1_	R_2_
Delphinidin 3-*O*-glucoside	OH	OH
Cyanidin 3-*O*-glucoside	OH	H
Petunidin 3-*O*-glucoside	OCH_3_	OH
Peonidin 3-*O*-glucoside	OCH_3_	H
Malvidin 3-*O*-glucoside	OCH_3_	OCH_3_

**Table 1:** Anthocyanins found in Cabernet Sauvignon red wine.

Peak No.	Compounds	Rt (min)	λmax	Precursor Ion	Product Ion	Concentration (mg/L)
1	Delphinidin 3-*O*-glucoside	7.978	524	465	303	2.801 ± 0.011
2	Cyanidin 3-*O*-glucoside	11.297	524	449	287	0.814 ± 0.010
3	Petunidin 3-*O*-glucoside	13.226	524	479	317	5.047 ± 0.014
4	Peonidin 3-*O*-glucoside	17.137	523	463	301	2.295 ± 0.001
5	Malvidin 3-*O*-glucoside	18.921	276, 525	493	331	137.815 ± 0.396
6	Vitisin A	22.183	510	561	399	6.743 ± 0.015
7	Vitisin B	24.669	-	517	355	4.106 ± 0.032
8	Malvidin 3-*O*-(6-*O*-acetyl)-glucoside-pyruvic acid	26.168	514	603	399	5.916 ± 0.026
9	Malvidin 3-*O*-glucoside-ethyl-catechin (1)	29.29	525	809	357	1.999 ± 0.357
10	Malvidin 3-*O*-(6-*O*-acetyl)-glucoside-acetaldehyde	29.926	495	559	355	4.012 ± 0.032
11	Malvidin 3-*O*-glucoside-ethyl-catechin (2)	30.847	532	809	357	0.337 ± 0.008
12	Malvidin 3-*O*-glucoside-ethyl-catechin (3)	32.762	532	809	357	0.404 ± 0.008
13	Peonidin 3-*O*-(6-*O*-acetyl)-glucoside	34.396	523	505	301	3.530 ± 0.464
14	Malvidin 3-*O*-(6-*O*-acetyl)-glucoside	35.799	528	535	331	80.068 ± 0.419
15	Malvidin 3-*O*-(6-*O*-*p*-coumaryl)-glucoside-pyruvic acid	36.989	517	707	399	1.382 ± 0.018
16	Malvidin 3-*O*-(6-*O*-caffeoyl)-glucoside	40.239	531	655	331	1.510 ± 0.382
17	Malvidin 3-*O*-(6-*O*-*p*-coumaryl)-glucoside-acetaldehyde	42.046	-	663	355	1.624 ± 0.208
18	Malvidin 3-*O*-(6-*O*-*cis*-*p*-coumaryl)-glucoside	42.615	532	639	331	1.354 ± 0.034
19	Peonidin 3-*O*-(6-*O*-*trans*-*p*-coumaryl)-glucoside	45.814	524	609	301	1.175 ± 0.433
20	Malvidin 3-*O*-(6-*O*-*trans*-*p*-coumaryl)-glucoside	46.45	531	639	331	16.081 ± 0.570
21	Malvidin 3-*O*-glucoside-4-vinylphenol adduct	-	-	609	447	-
22	Malvidin 3-*O*-(6-*O*-acetyl)-glucoside-4-vinylphenol adduct	-	-	651	447	-
	Total anthocyanins	-	-	-	-	299.752 ± 2.352

Note: “-”Not detected.

**Table 2:** Phenolic acids found in Cabernet Sauvignon red wine.

Peak No.	Compounds	Rt (min)	λmax	Concentration (mg/L)
1	Gallic acid	4.243	271	11.676 ± 0.321
2	Protocatechuic acid	7.319	259, 293	4.771 ± 0.208
3	Vanillic acid	15.363	291	8.156 ± 0.853
4	Syringic acid	16.671	274	21.350 ± 0.412
5	*p*-Coumaric acid	26.818	309	2.846 ± 0.162

**Table 3:** Degradation products from malvidin 3-*O*-glucoside standard under the simulated digestion process.

Compound	λmax	Precursor Ion	Product Ion
Syringic acid	274, 279	197	153
Peonidin 3-*O*-glucoside *	-	463	301
Malvidin 3-*O*-glucoside *	525	493	331
Malvidin 3-*O*-glucoside chalcone *	343	511	349, 223
Malvidin 3-*O*-glucoside chalcone	343	509	347, 221

Note: * Positive electrospray ionization mode.
